# How combining different caries lesions characteristics may be helpful in short-term caries progression prediction: model development on occlusal surfaces of primary teeth

**DOI:** 10.1186/s12903-021-01568-2

**Published:** 2021-05-12

**Authors:** Isabela Floriano, Elizabeth Souza Rocha, Ronilza Matos, Juliana Mattos-Silveira, Kim Rud Ekstrand, Fausto Medeiros Mendes, Mariana Minatel Braga

**Affiliations:** 1 Department of Orthodontics and Paediatric Dentistry, School of Dentistry, University of Sao Paulo, Avenida Professor Lineu Prestes, 2227, Cidade Universitária, São Paulo, SP Brazil; 2 Dentistry Course, University Uninovafapi Centre, Teresina, Piaui Brazil; 3 Dental School, Fundação Hermínio Ometto, Araras, Sao Paulo, Brazil; 4 Section of Cariology and Endodontics, Dental School of Copenhagen, University of Copenhagen, Copenhagen, Denmark

**Keywords:** Dental caries, Caries activity, Validation studies, Primary teeth, Visual inspection

## Abstract

**Background:**

Few studies have addressed the clinical parameters' predictive power related to caries lesion associated with their progression. This study assessed the predictive validity and proposed simplified models to predict short-term caries progression using clinical parameters related to caries lesion activity status.

**Methods:**

The occlusal surfaces of primary molars, presenting no frank cavitation, were examined according to the following clinical predictors: colour, luster, cavitation, texture, and clinical depth. After one year, children were re-evaluated using the International Caries Detection and Assessment System to assess caries lesion progression. Progression was set as the outcome to be predicted. Univariate multilevel Poisson models were fitted to test each of the independent variables (clinical features) as predictors of short-term caries progression. The multimodel inference was made based on the Akaike Information Criteria and C statistic. Afterwards, plausible interactions among some of the variables were tested in the models to evaluate the benefit of combining these variables when assessing caries lesions.

**Results:**

205 children (750 surfaces) presented no frank cavitations at the baseline. After one year, 147 children were reassessed (70%). Finally, 128 children (733 surfaces) presented complete baseline data and had included primary teeth to be reassessed. Approximately 9% of the reassessed surfaces showed caries progression. Among the univariate models created with each one of these variables, the model containing the surface integrity as a predictor had the lowest AIC (364.5). Univariate predictive models tended to present better goodness-of-fit (AICs < 388) and discrimination (C:0.959–0.966) than those combining parameters (AIC:365–393, C:0.958–0.961). When only non-cavitated surfaces were considered, roughness compounded the model that better predicted the lesions' progression (AIC = 217.7, C:0.91).

**Conclusions:**

Univariate model fitted considering the presence of cavitation show the best predictive goodness-of-fit and discrimination. For non-cavitated lesions, the simplest way to predict those lesions that tend to progress is by assessing enamel roughness. In general, the evaluation of other conjoint parameters seems unnecessary for all non-frankly cavitated lesions.

**Supplementary Information:**

The online version contains supplementary material available at 10.1186/s12903-021-01568-2.

## Background

Active enamel caries lesions have been generally defined as those that present rough enamel and loss of lustre (indicated by chalky-white enamel) [1, 2]. However, differential features linked to enamel caries activity status have been attributed to differences in enamel porosity and surface wear/polishing [3, 4]. These clinical features have traditionally been observed under specific conditions (areas of intense plaque accumulation and short-term remineralization of enamel lesions) and on particular surfaces [2]. Although the characteristics that guide caries lesion activity assessment are strongly intercorrelated [5], we hypothesized that some of the characteristics could be more closely related to caries progression than others, particularly considering the occlusal surfaces.

Available visuotactile systems for caries lesion activity assessment recommend that caries lesions' clinical features should be conjointly considered because caries is a dynamic process [6, 7]. Previous studies have verified the predictive validity of these systems [8, 9]. However, because these systems propose the conjoint evaluation of a pool of clinical characteristics detected through visuotactile inspection, we cannot affirm the predictive power of individual clinical features associated with the status of caries lesions. This study is the first to prospectively evaluate each clinical characteristic's influence on caries lesion progression and propose a simpler predictive model to guide clinical decision making even in a short-term analysis by distinguishing those caries lesions that demand immediate management. Otherwise, they can progress and require dental restorations.

Predictive validation has been indicated as the optimal choice for determining the validity of the assessment of caries lesion activity [10]. Also, it determines how well a test (in this case, individual components of available systems) can predict future events [11] as caries progression. The predictive models have been extensively useful in medical practice to screen for diseases, establish diagnosis and prognosis to guide therapeutical decision-making, and inform patients about the possible natural history of certain conditions or prognoses after some intervention received [12]. Commonly, quite complex health-related multivariate models are developed and tested [13, 14]. From other areas of knowledge, we can observe that the most complex models better predict an outcome of interest [15]. However, in the present study, the idea was to simplify the model prediction for caries progression. Accordingly, we aimed to derive a predictive model for short-term (1 year) progression of caries lesions on occlusal surfaces of primary molars using only characteristics based on lesions assessment/detection and not the child’s  characteristics associated to this outcome.

## Methods

According to the Transparent Reporting of a prediction model for Individual Prognosis or Diagnosis (TRIPOD), this manuscript has been prepared for reporting prediction model proposal/development focused on the short-term progression of caries lesions.

### Examiner training

Two examiners were involved in this study: one responsible for the baseline examinations (MMB) and the other for the follow-up (IF). This last examiner (IF) was introduced to the ICDAS by an experienced examiner (MMB), who is engaged in previous clinical studies in caries diagnosis. This experienced examiner was considered as a reference examiner for this study. Firstly, the original index description was studied [16]. The trained examiner then individually evaluated projections of clinical photographs of caries lesions. Finally, 36 occlusal surfaces of extracted primary molars were assessed for both separately. Among these surfaces, sound surfaces and caries lesions at different levels of severity were included. After each training phase, the reference examiner discussed any divergence between the examiners and coordinated the training. The next step was started after all doubts and divergences had been solved.

For the assessment of potential predictors, the reference examiner examined this same sample of teeth twice in random orders to permit the calculation of intra-examiner calibration.

### Source of data/study population/participant selection

The focus of this prospective cohort study was on children with primary molars (approximately from 3 to 12 years) who sought dental treatment at a dental clinic. These children could have preventive or therapeutic needs (caries experience or not). The clinic, located in a Dental School in São Paulo, Brazil, is a reference for Pediatric Dentistry. São Paulo presents human development index equal to 0.805 (https://www.br.undp.org/content/brazil/pt/home/idh0/rankings/idhm-municipios-2010.html) and regularly fluoridated water at 0.7 ppm. This Brazilian region's mean dmft index among 5-year-old children is 2.1 (95%CI 1.79–2.42) [17].

This sample constituted our development cohort. At this time, we did not include a validation cohort. This cohort was formed from 2011 to 2013 and then was followed up for one year. Children from this cohort were referred to dental care in the same dental school clinics where the research was being conducted. However, neither the researchers were not responsible for their treatment, nor specific research protocol for dental treatment was adopted for these children. Protocols used for dental care providers at the institution (or where they had the dental treatment performed) were followed independently of research participation.

All children who had at least one primary molar available to be examined were eligible for enrolment. The children's assent and their parents' consent had to be obtained to guarantee participation in the study. When consent/assent was not obtained, the child was excluded from the sample. The same was done when children and their family stated that it is impossible for them to comply with the 1-year follow up.

If a child presented more than one eligible molar, one included all of them. Surfaces with restorations, hypoplastic defects, sealants, or frank cavities (ICDAS 5–6) were excluded from the study sample.

An external researcher pre-selected a site for each surface based on the highest ICDAS score found on the respective surface. Sites were recorded using a specific illustration in the participant's file to guide the following stages.

### Study outcome

Caries progression was set as the primary outcome. As caries progression, we considered those surfaces that, with a 1-year follow-up, presented cavities with dentine exposure and/or teeth were restored or extracted due to caries. This examination was performed approximately one year after the baseline examination by a different examiner from the baseline assessments. On this occasion, children occlusal surfaces were examined using the ICDAS [16]. Restorations and teeth that had been extracted (because of caries) were also recorded. Changes not related to cavitation exposing the dentine (e.g., ICDAS score 1 or 2 to ICDAS 3) were registered, but they were not considered as an event for the analysis.

The children were examined in a dental unit using the halogen operating light lamp for the dental chair. Examiner used a plane dental mirror, a ball-ended probe, and a three-in-one syringe. Before the examination, teeth were gently cleaned with a rotating bristle brush and pumice/water slurry. For this evaluation, the examiner followed pre-signalled charts with the occlusal sites evaluated at the baseline.

### Sample size

The required sample size was estimated based on the assessment of active caries lesions in children. Sample size calculation [18] was based on a prevalence of active lesions of 62.5%, observed in a previous study conducted on a Brazilian population [19]. We assumed that surfaces with active caries lesions would be those prone to progress for frankly cavitated lesions. For this calculation, we adopted the most conservative condition to guarantee the maximum possible sample size, as if one surface could be included per child and a confidence level of 95%. A minimum sample size of 126 surfaces was calculated, and this number was increased by 20% to compensate for parent or children's refusal to participate and for possible dropouts. Hence, a sample of 151 surfaces was required. Since more than one occlusal surface could be included in the sample, we assumed a factor of correction of 1.4 to compensate for the clustering effect. Then, we determined that at least 212 surfaces were needed for our sample.

### Possible predictor variables

**Fig. 1:**
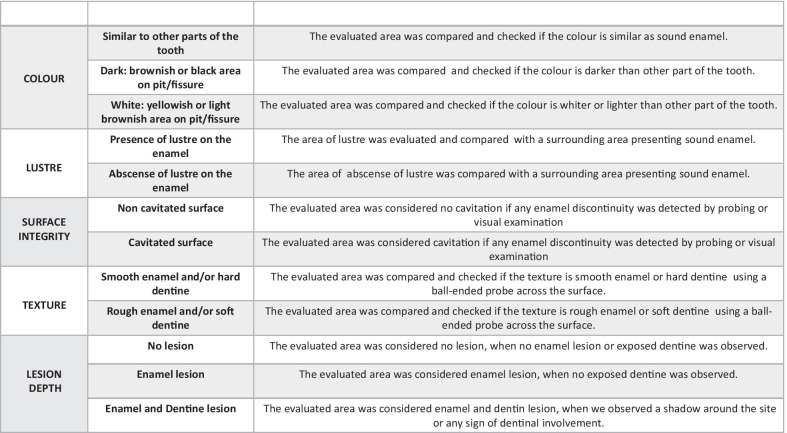
Description of the clinical assessment of possible predictor variables

We identified possible predictor variables based on previously published literature review [2] describing possible clinical parameters associated with active caries [5]. These parameters have been combined in different systems for caries activity assessment [7, 20] and were described in details in a previous publication [5] (Fig. 1). In the present study, we decided to investigate these parameters' ability in predicting short-term caries progression (1 year). For that, we tested their prediction solely or in combination with one or more parameters under investigation.

An experienced examiner in caries diagnostic research (MMB), different from that of the follow-up examination, assessed eligible occlusal surfaces in the baseline and classified the selected sites by independently considering the possible predictor variables: colour, lustre, surface integrity, depth, and texture (Fig. 1). The examiner did not use any specific system but classified the sites as described. The clinical examination was performed at the same clinical conditions (light, air-drying, and the use of specific probe) mentioned above for outcome assessment.

### Model proposal/derivation

For model development, we opted for a complete-case analysis, excluding those cases in which there is the absence of information related to the prediction variables assessment and/or follow-up assessment for outcome evaluation. Firstly, each of the clinical features related to caries lesions' activity status (colour, lustre, surface integrity, texture, and lesion depth) was tested as independent variables (Models 1–7). Univariate multilevel Poisson models were fitted to test each of the independent variables (clinical features) as predictors of short-term caries progression. These variables were chosen based on information criteria [21], and plausible models were tested. Since there is a previously established theory supporting the studied construct, we opted to perform a multimodel inference based on the Akaike Information Criteria (AIC) [21, 22]. The unit of analysis was the tooth. The levels for these analyses were: the tooth and the child. This option permits the adjustment of analyzing more than one tooth per child, if necessary.

**Fig. 2:**
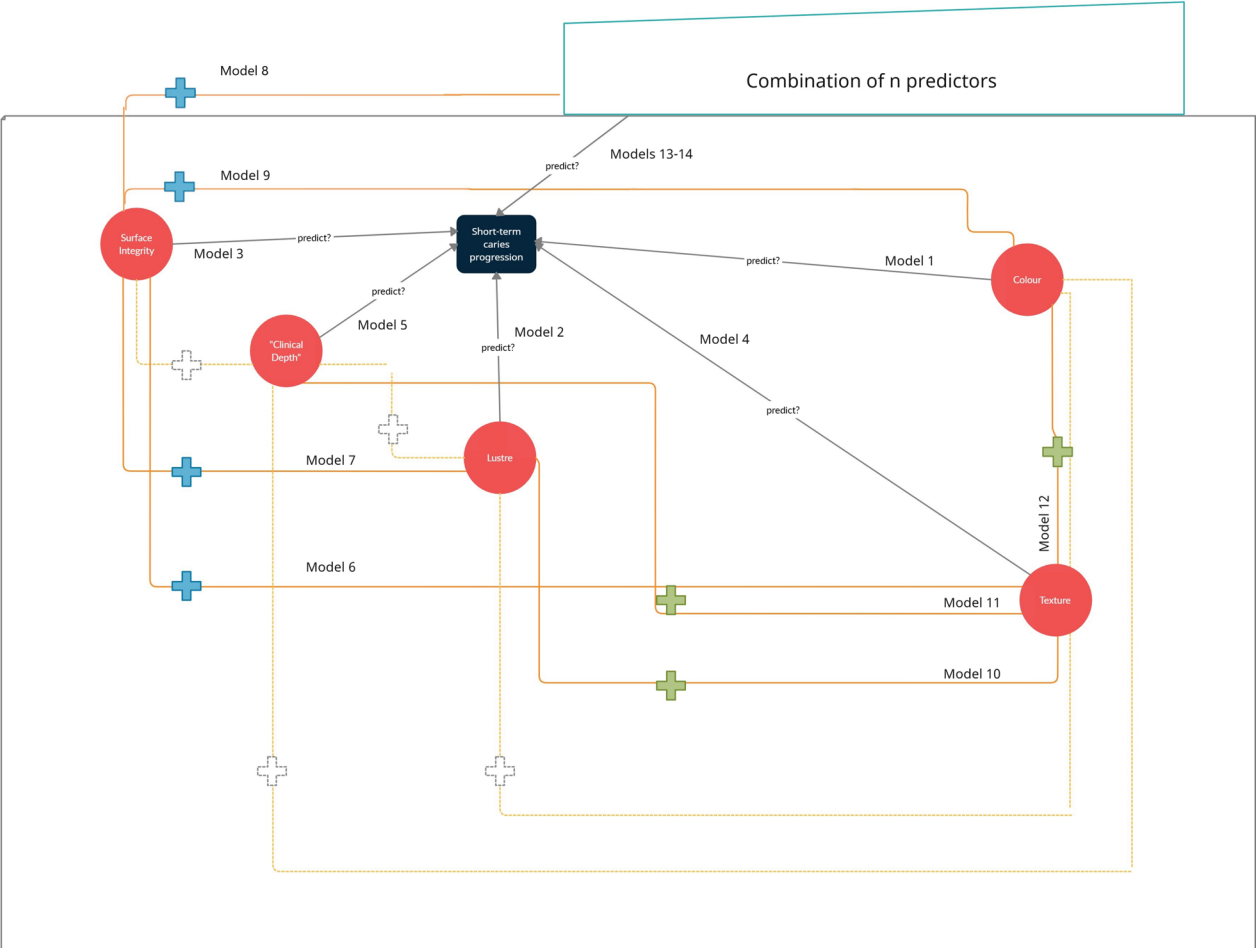
The conceptual and statistical framework of potential interactions tested in the predictive models. Arrows indicate the direction of prediction. Red circles represent the predictor (lesion characteristic) used in univariate models (#1–#5). Signal “+” symbolizes the interaction of individual variables or combination using dummy variables. Solid lines/symbols indicate the interactions considered for modelling considering a priori conceptual framework and the statistical appreaciation of AIC (Models #6–#13). Predictors with lower AICs-surface integrity (orange symbols) and texture (green symbols)- were combined to produce interactions with other relevant predictors to permit estimating their combined effect on predicting caries progression. Dashed lines/symbols indicate those interactions which could potentially exist in the conceptual framework, but they were not presented in our Results for statistical options guided by the AIC values

Afterwards, we tested the plausible interactions among some variables to evaluate the possible benefit of combining these variables when assessing caries lesion activity, as proposed for some available systems. The conceptual and statistical framework of these potential interactions is illustrated in Fig. 2. We tested the interactions between two variables using the interaction terms (product variables) created as dummy variables in the respective models (Additional file 1). Firstly, these “product terms” were used to represent the interaction between variables based on a priori meaning defined in the conceptual framework (Fig. 2). Moreover, variables resulting in lower AICs in univariate models were first selected to be tested in conjunction with the others (statistical framework—Fig. 2) since a reasonable construct was available. All possible combinations were tested, but only the most relevant will be described in the results.

The dummy variables created were used as independent variables in the regression equation of multilevel models and tested as a meaningful predictor (Additional file 2). Hence, it is used to investigate the potential benefit of combining two variables when predicting the outcome of interest. A multiple regression model was not used including more than one characteristic as independent variables because of the likelihood of multicollinearity among them. Indeed, one variable could cancel out the effect of another related variable in the multiple model if they are strongly associated with each other, even being equally crucial individually in explaining the outcome.

Finally, to simulate the use of systems that combine these characteristics, we created specific dummy variables representing the number of active lesions' characteristics. First, we assumed an initial or established active lesion would be ideally whitish/yellowish with no lustre and rough enamel. In one of the models, we tested if occlusal surfaces, presenting at least two of these positive factors, may predict caries progression after one year. For the other one, we tested if the presence of any one of the positive factors above could predict the same outcome (Additional file 3).

Subgroup analyses were also performed by considering only the non-cavitated lesions at the baseline. We adopted the same strategies mentioned earlier for model derivation for these analyses, but the interpretation of results was made carefully due to the limitations inherent to this approach.

### Prediction model performance

The relative risk (RR) for the clinical features predicting the outcome (alone or combined with one or more other clinical features) was calculated with a 95% confidence interval (95% CI). Two-sided p values < 0.05 were considered to be statistically significant.

The overall goodness-of-fit of the models was compared in the development cohort based on AIC. To evaluate model discrimination, we used the C statistic [12]. As we had a dichotomous outcome, we calculated the area under Receiver Operator Characteristic (ROC) curves to obtain the C-statistics [12].

We used bootstrap resampling for internal validation to adjust for the overfitting and optimistic performance of the model. One thousand bootstrap samples were drawn with replacement, and the performance was also evaluated in the bootstrap sample. The uniform shrinkage factor was computed using this bootstrap procedure [23], and regression slopes were recalculated based on that.

Statistical analyses were performed in Stata Software (version 13.1), StataCorp, Texas, USA.

## Results

The intra-examiner reproducibility in the assessment of clinical parameters by the experienced examiner was high (Kappa values: 0.93 (95% CI 0.90–0.97) for lesion depth and surface integrity, 0.98 (95% CI 0.97–1.0) for colour, 0.88 (95% CI 0.85–0.91) for texture, and 0.90 (95%CI 0.87–0.93) for luster). Regarding the follow-up examiner, intra-examiner agreement (weighted Kappa) of 0.849 and inter-examiner agreement of 0.92 were reached considering the experienced examiner as reference.

**Fig. 3:**
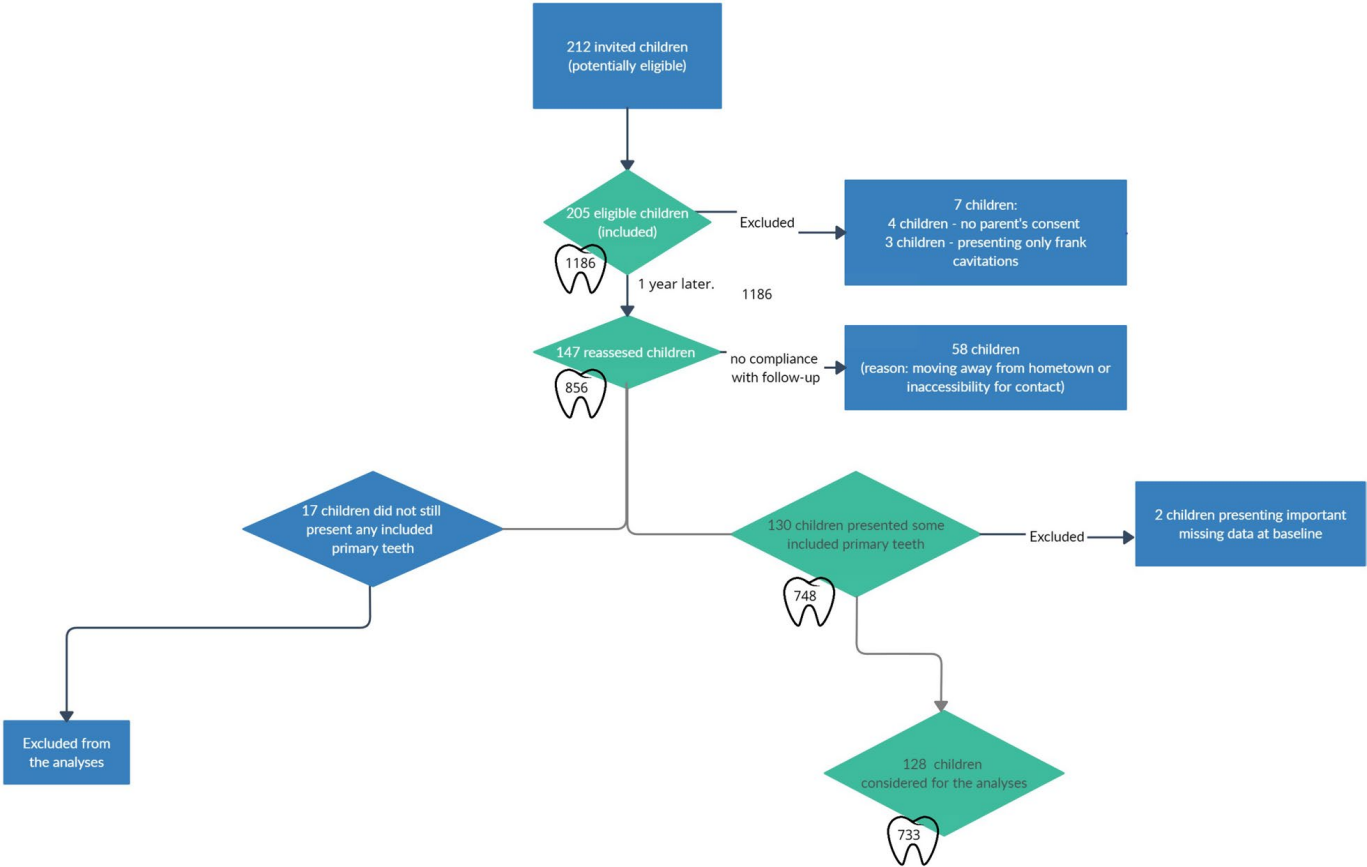
Flowchart of the participant's selection and follow-up in the study

Two hundred and twelve children were invited to participate in the study. Seven children could not be included. Four parents refused to join because they would not comply with the follow-up for one year. Three children were actually not eligible because they only have frank cavities on their primary molars' occlusal surfaces. Out of 205 children examined at the baseline, 1189 occlusal surfaces of primary molars (comprising ICDAS scores from 0 to 4), 100 were girls (49%) and 105 were boys (51%). The mean age (standard deviation, SD) was 7 (2.1) years. After approximately one year, 147 children (71%) were reassessed (Fig. 3). 748 occlusal surfaces (63%) could be evaluated regarding caries progression in a 1-year follow-up (Fig. 3). 108 surfaces (9%) were unavailable for evaluation because the primary teeth had already exfoliated. 16 surfaces (0.01%) had not been assessed with different clinical parameters related to caries lesions at the baseline. Due to that, they were not considered for analysis. A total of 733 surfaces (61%) were finally analyzed (Fig. 2). The mean time of re-examination (SD) was 395 (70.8) days.

The children reassessed after one year had similar caries experience based on the decayed, missing, and filled surfaces index (mean dmfs + DMFS = 3.28; 95%CI 2.20 to 4.36) when compared with those who were not followed-up in the study (mean dmfs + DMFS  = 3.36; 95%CI 2.25 to 4.46, p = 0.35). 8.5% of evaluated occlusal surfaces (n = 62) was presented with caries progression after 1 year (31 presented frank cavitations, 28 had been restored, and 3 had to be extracted due to caries). Tables 1 and 2 show how surfaces differently scored at the baseline were found after 1 year.

**Table 1:** Classification of the surfaces (n) at baseline, according to the clinical parameters, and at 1-year follow-up, according to the ICDAS

Clinical predictors	1-year follow-up assessment (ICDAS codes)									
	0	1	2	3	4	*5*	*6*	*EX*	*R*	Total
Color										
No staining	262	55	6	10	2	*4*	*3*	*1*	*4*	347
Whitish	8	33	11	25	10	*8*	*1*	*1*	*8*	105
Yellowish	36	52	21	30	2	*2*	*2*		*4*	149
Black or dark brownish	13	27	14	48	6	*10*	*1*	*1*	*12*	132
Lustre										
Present	284	100	18	51	10	*13*	*5*	*2*	*11*	494
Absent	35	67	34	62	10	*11*	*2*	*1*	*17*	239
Surface integrity										
Non-cavitated	316	155	46	73	7	*9*	*7*	*1*	*13*	627
Cavitated	3	12	6	40	13	*15*		*2*	*15*	106
Texture										
Smooth enamel	288	112	20	51	6	*10*	*4*	*1*	*12*	504
Rough enamel	31	55	32	62	14	*14*	*3*	*2*	*16*	229
"Clinical depth"										
Sound	260	56	6	10	1	*4*	*3*	*1*	*4*	345
Enamel	59	109	44	97	12	*13*	*3*	*2*	*13*	352
Dentine*		2	2	6	7	*7*	*1*		*11*	36
Total	319	167	52	113	20	*24*	*7*	*3*	*28*	733

0–6 = ICDAS scores; EX = indicated for extraction/extracted tooth due to caries; R = restored surface. * Lesions were clinically classified into dentine only if a shadow was observed under enamel (even without dentine exposure)—cavities exposing dentine were not considered in these analyses

The italics values highlighted columns correspond to the surfaces on which were considered progression

**Table 2:** Short-term caries progression after 1 year (n (%)) for different clinical predictors

All surfaces			
Colour			
No staining	335 (97%)	12 (3%)	347
Whitish	87 (83%)	18 (17%)	105
Yellowish	141 (95%)	8 (5%)	149
Black or dark brownish	108 (82%)	24 (18%)	132
Lustre			
Presence	463 (94%)	31 (6%)	494
Absence	208 (87%)	31 (13%)	239
Surface integrity			
Non-cavitated	597 (95%)	30 (5%)	627
Cavitated	74 (69%)	32 (31%)	106
Texture			
Smooth enamel	477 (95%)	27 (5%)	504
Rough enamel	194 (84%)	35 (16%)	229
"Clinical depth"			
No lesion	333 (97%)	12 (3%)	345
Enamel lesion	321 (91%)	31 (9%)	352
Dentine Lesion	17 (47%)	19 (53%)	36
Total	671 (92%)	62 (8%)	733

Non-cavitated surfaces			
Colour			
No staining	335 (96%)	12 (4%)	347
Whitish	59 (91%)	6 (9%)	65
Yellowish	128 (95%)	7 (5%)	135
Black or dark brownish	75 (94%)	5 (5%)	80
Lustre			
Presence	425 (96%)	18 (4%)	443
Absence	172 (94%)	12 (6%)	184
Texture			
Smooth enamel	449 (96%)	17 (4%)	466
Rough enamel	148 (92%)	13 (8%)	161
"Clinical depth"			
No lesion	333 (97%)	12 (3%)	345
Enamel lesion	261 (94%)	16 (6%)	277
Dentine Lesion	3 (60%)	2 (40%)	5
Total	597 (95%)	30 (5%)	627

Except for the lustre, all variables tested were associated with the short-term caries progression in one year (Table 3). Among the univariate models created with each one of these variables, the model containing the surface integrity as a predictor had the lowest AIC (Model 3, AIC = 364.5). The second best parameter to predict caries progression was clinical depth (Model 5, AIC = 369.7). Models in which variables interactions or combinations (Models 6 to 3) were tested did not present lower AICs. C statistics were quite similar for univariate models testing both variables individually or their interactions/combinations (Table 2). In the internal validation cohort, all tested models remained well-calibrated (Additional file).

**Table 3:** Relative risks (RR) with 95% confidence interval (95% CI), goodness-of-fit (Aikaike Inference Criteria - AIC) and discrimination (C statistic/area under ROC curve - AUC) of univariate models for predicting caries progression (cavitation—ICDAS 5 or 6, restoration or the tooth extraction due to caries) on occlusal sites examined, followed by one year—section A: all non-frankly cavitated surfaces (ICDAS scores 0–4) at the baseline, section B: only non-cavitated surfaces (ICDAS 0–2) included at the baseline

		All surfaces				
	Null Model	Model 1	Model 2	Model 3	Model 4	Model 5
	RR (95%IC)	RR (95%IC)	RR (95%IC)	RR (95%IC)	RR (95%IC)	RR (95%IC)
Colour						
ref. Similar to sound						
Whitish fissure		5.17 (2.21 to 12.11)				
Yellowish fissure		1.42 (0.55 to 3.69)				
Black or dark fissure		3.48 (1.60 to 7.54)				
Lustre						
ref. Presence						
Absence			1.70 (0.98 to 2.96)			
Surface integrity						
ref. Noncavitated						
Cavitated				4.95 (2.78 to 8.80)		
Texture						
ref. Smooth enamel						
Rough enamel					2.15 (1.19 to 3.91)	
"Clinical" depth						
ref. Sound						
Enamel						2.27 ( 1.12 to 4.63)
Dentine						9.50 (3.98 to 22.68)
_ cons	0.03 (0.02 to 0.06)	0.19 (0.88 to 1.96)	0.03 (0.01 to 0.05)	0.03 (0.02 to 0.05)	0.03 (0.01 to 0.05)	0.02 (0.01 to 0.04)
Random effects parameters						
Estimate (95%CI)						
Patient- sd (cons)	1.58 (1.10 to 2.72)	1.31 (0.87 to 1.96)	1.48 (1.02 to 2.14)	1.15 (0.76 to 1.75)	1.39 (0.94 to 2.04)	1.06 (0.67 to 1.68)
Model goodness-of-fit and Discrimination						
AIC	389.4624	376.8412	387.96460	364.7552	385.2818	369.7105
C statistic (AUC)	0.9653	0.9609	0.9651	0.9607	0.9661	0.9591
95%CI	0.9516 to 0.9790	0.9407 to 0.9810	0.9513 to 0.9789	0.9437 to 0.9778	0.9530 to 0.9791	0.9405 to 0.9776

		Non cavitated surfaces (subgroup analysis)				
	Null Model	Model 1	Model 2	Model 3	Model 4	Model 7
	RR (95%IC)	RR (95%IC)	RR (95%IC)	RR (95%IC)	RR (95%IC)	RR (95%IC)
Color						
ref. Similar to sound						
Whitish fissure		3.06 (0.93 to 10.07)				
Yellowish fissure		1.39 (0.49 to 3.93)				
Black or dark fissure		1.19 (0.39 to 3.65)				
Lustre						
ref. Presence						
Absence			1.66 (0.73 to 3.80)			
Surface integrity						
ref. Noncavitated						
Cavitated				–		
Texture						
ref. Smooth enamel						
Rough enamel					1.95 (0.85 to 4.47)	
"Clinical" depth						
ref. Sound						
Enamel						1.40 ( 0.62 to 3.20)
Dentine						8.87 (0.98 to 80.15)
_ cons	0.01 (0.004 to 0.04)	0.01 (0.003 to 0.03)	0.01 (0.003 to 0.03)	–	0.01 (0.03 to 0.03)	0.01 (0.004 to 0.04)
Random effects parameters						
Estimate (95%CI)						
Patient- sd (cons)	1.86 (1.11 to 3.11)	1.78 (1.06 to 2.99)	1.80 (1.08 to 3.00)	–	1.76 (1.06 to 2.96)	1.62 (0.97 to 2.75)
Model goodness-of-fit and discrimination						
AIC	218.1902	221.0009	218.80930	not applicable	217.7614	218.84
C statistic (AUC)	0.9160	0.9164	0.8983	not applicable	0.9081	0.9519
95%CI	0.8827 to 0.9496	0.8793 to 0.9536	0.8536 to 0.9429		0.8695 to 0.9467	(0.9405 to 0.9775)

The lowest AICs observed for models testing interactions (better goodness-of-fit) were those in which surface integrity was combined with any other clinical parameter (Models 6 to 9—Table 4). However, AICs were still higher when the surface integrity was tested solely (Model 3—Table 4). Generally, in assessing non-frankly cavitated lesions, detecting surface discontinuity may better predict if this lesion will progress in one year than considering other related clinical parameters. In the development cohort, cavitated lesions presented approximately a five-fold higher risk for short-term caries progression than non-cavitated surfaces (Tables 2 and 3). Dentine lesions had a probability of progression approximately ten times higher than that of sound surfaces. Conversely, enamel lesions were almost 3 times more prone to progression than sound sites (Tables 2 and 3).

**Table 4:** Relative risks (RR) with 95% confidence interval (95% CI), goodness-of-fit (Aikaike Inference Criteria - AIC) and discrimination (C statistic - Area under of ROC curve - AUC) of models for predicting caries progression (cavitation—ICDAS 5 or 6, restoration or the tooth extraction due to caries) after one year considering the combination of clinical features related to caries lesion activity status on examined occlusal surfaces

All lesions										
		Combinations with surface integrity				Combinations with texture			Combinations of n parameters (irrespective which)	
	Null Model	Model 6	Model 7	Model 8	Model 9	Model 10	Model 11	Model 12	Model 13	Model 14
	RR (95%IC)	RR (95%IC)	RR (95%IC)	RR (95%IC)	RR (95%IC)	RR (95%IC)	RR (95%IC)	RR (95%IC)	RR (95%IC)	RR (95%IC)
Texture + Depth										
Ref. Smooth surface + No lesion										
Smooth surface + Enamel lesion							1.81 (0.75 to 4.38)			
Smooth surface + Dentine lesion							9.39 (2.72 to 32.24)			
Rough surface + Enamel lesion							2.63 (1.22 to 5.64)			
Rough surface + Dentine lesion							9.84 (3.80 to 25.50)			
Color + Texture										
ref. No color + smooth area										
Whitish + smooth area								3.28 (1.12 to 9.64)		
Yellowish + smooth area								1.59 (0.41 to 6.09)		
Dark brownish/black + smooth area								2.81 (0.96 to 8.20)		
Whitish + rough area								7.58 (2.80 to 19.90)		
Yellowish + rough area								1.37 (0.45 to 4.16)		
Dark brownish/black + rough area								4.12 (1.79 to 9.48)		
Cavitation + Texture										
ref. Non-cavitated + smooth area										
Non-cavitated + rough area		2.06 (0.95 to 4.44)								
Cavitated + smooth area		6.64 (2.79 to 15.81)								
Cavitated + rough area		6.55 (3.08 to 13.95)								
Texture + Lustre										
ref. Smooth area with lustre										
Smooth area without lustre						2.85 (1.15 to 7.05)				
Rough area with lustre						3.46 (1.49 to 8.06)				
Rough area without lustre						2.40 (1.21 to 4.75)				
Cavitation + Lustre										
Non-cavitated with lustre										
Non-cavitated without lustre			1.61 (0.75 to 3.49)							
Cavitated with lustre			5.72 (2.56 to 12.70)							
Cavitated without lustre			6.06 (2.91 to 12.63)							
Color + Cavitation										
ref. No color + smooth area										
Whitish + smooth area					3.07 (1.07 to 8.79)					
Yellowish + smooth area					1.45 (0.55 to 3.85)					
Dark brownish/black + smooth area					1.49 (0.50 to 4.41)					
Whitish + rough area					8.98 (3.47 to 23.27)					
Yellowish + rough area					2.37 (0.28 to 20.11)					
Dark brownish/black + rough area					6.86 (3.00 to 15.67)					
Cavitation + Other signs for activity*										
Non-cavitated + No other activity sign or only one activity sign										
Non-cavitation + Opaque + Rough + No dark area				0.74 (0.29 to 1.89)						
Non-cavitated + No other activity sign or only one sign activity sign				1.60 (0.33 to 7.72)						
Cavitated + Opaque + Rough + No dark area				2.00 (0.60 to 6.62)						
None present positive factor										
1 positive factor									3.26 (1.49 to 7.15)	
At least 2 positive factors									3.50 (1.61 to 6.95)	
Number of factor combined (Lustre/Texture/Color)										
None present positive factor										3.13 (1.67 to 6.57)
At least 1 positive factor										
_ cons	0.03 (0.02 to 0.06)	0.02 (0.01 to 0.04)	0.02 (0.01 to 0.05)	0.03 (0.02 to 0.06)	0.02 (0.01 to 0.04)	0.02 (0.01 to 0.05)	0.02 (0.01 to 0.05)	0.02 (0.009 to 0.04)	0.02 (0.008 to 0.04)	0.02 (0.92 to 1.97)
Random effects parameters										
Patient- sd (cons)	1.58 (1.10 to 2.72)	1.12 (0.74 to 1.71)	1.13 (0.75 to 1.71)	1.54 (1.06 to 2.23)	1.13 (0.74 to 1.73)	1.30 (0.88 to 1.93)	1.05 (0.66 to 1.67)	1.23 (0.82 to 1.87)	1.34 (0.92 to 1.97)	1.35 (0.92 to 1.97)
Model goodness-of-fit and Discrimination										
AIC	389.4624	365.49730	367.28350	393.43680	369.10040	383.82820	372.8669	380.19200	380.21180	378.21840
C statistic (AUC)		0.9616	0.9619	0.9612	0.9581	0.9631	0.9560	0.9606	0.9622	0.9651
		0.9444 to 0.9788	0.9446 to 0.9792	0.9411 to 0.9814	0.9389 to 0.9773	0.9473 to 0.9789	0.9359 to 0.9762	0.9495 to 0.9806	0.9430 to 0.9814	0.9514 to 0.9788

When the surface integrity was not considered, clinical depth and texture were the best interaction of clinical parameters (Model 11, AIC = 372.8). Indeed, when considering only non-cavitated lesions, dentine lesions (shadows) tended to maintain their association with short-term lesion progression (RR = 8.9; 95%CI 0.98–80.15—p = 0.052). However, very few dentine non-cavitated lesions were included in the sample (Table 2).

On the other hand, models considering the texture (Model 3, AIC = 217.7), or at least two any factors positive for activity as predictors for short-term caries progression (Model 13, AIC = 217.8), were those who presented the highest goodness-to-fit when predicting progression among the non-cavitated lesions. Both rough lesions (RR = 1.93; 95% CI 0.85–4.47) or those with two positive factors for activity (RR = 1.93; 95% CI 0.85–4.39) were not statistically associated with caries progression, but they tended to present a 90% higher risk of progressing in 1-year time (Table 3, section B). Given the limitations of subgroup analysis for non-cavitated surfaces, assessing its roughness may help to distinguish those lesions prone to short-term progress. Alternatively, clinicians may use two positive parameters for activity, including rough texture, whitish colour and absence of lustre, to make this prediction.

## Discussion

Available visuotactile systems for caries lesion activity assessment recommend evaluating caries lesions' clinical features conjointly [7, 20]. Nevertheless, the best prediction of short-term caries progression (1 year) was found when univariate models were used. When combinations of some clinical characteristics were prospectively assessed, additional contribution/benefit seems to be observed only in particular situations.

This study aimed to clarify the predictive power of different clinical characteristics of active lesions for predicting short-term caries progression. That is why the predictive model considering only features related to caries lesions was proposed. Our motivation was to guide clinicians about what they should assess to choose a therapeutic option for their patients when managing caries lesions. In this sense, clinicians might find a more accurate way of predicting those lesions that would progress in 1-year time and guide his/her decision-making. Other important patient-related variables are crucial for predicting caries progression in the broader sense, for example, caries experience [24, 25]. They are usually correlated/collinear with those parameters related to the lesions. However, intentionally, they were not included in the fitted models to evaluate the predictive power of the mentioned clinical characteristics.

Hence, we used a sample selected from children who had sought dental treatment. Since our sample was calculated a priori, we based it on a younger population [19]. Older children may have less active caries lesions [26]. Then, some lesions may have had more time and opportunity to be arrested. Accordingly, a larger sample would be necessary if we considered this difference. Conversely, the age group included may reflect a higher likelihood of seeking treatment, thereby representing the population we aimed to study. In general, we obtained statistical power for permitting to propose and explore models to predict short-term caries progression, even considering this limitation. Therefore, our results may be extrapolated to a population of children who seek treatment in dental clinics.

Our results evidenced the presence of cavities may be a decisive factor for predicting caries lesion progression. Approximately 30% of cavitated caries lesions progressed after one year. Even occlusal caries lesions related to microcavities (ICDAS 3)—clinically into the enamel—may be found histologically into dentine [27, 28]. A high infection level has also been found at the enamel–dentine junction [29]. Cavitated lesions are often histologically active [2, 30] and are more challenging to be arrested than non-cavitated lesions. The present prediction model, based exclusively on surface integrity, may help clinicians in private or public systems to minimally distinguish those lesions (even previous to frank cavitation) that should be prioritized when managing caries lesions.

Despite a strict association between dentine lesions and the presence of cavitation in primary teeth [31], the model prediction based on clinical depth presented inferior performance than the one based on surface integrity. Those dentine lesions worth being seen clinically are those presenting shadows. However, they are not so frequent in primary teeth [19, 32]. Furthermore, this type of lesion tends to be more challenging to be detected, corroborating the feasibility of assessing the presence of cavity (surface discontinuation) to predict short-term caries progression and indicate specific measures to stop this process.

Differently from established caries lesions for which severity may be sufficient for predicting progression, assessing some other features of non-cavitated caries lesions could be useful. Non-cavitated lesions, which presented at least two clinical features scored as positive for active lesions (whitish/yellowish color, loss of luster, or rough surface) [2] had a higher risk of progression than sites with no lesions. A slightly lower power of prediction was found when only one factor was considered. On the other hand, a similar prediction ability was found when the texture was considered solely.

The use of multiple combined parameters has been likely advocated since we are assessing a dynamic process, caries activity, and we can find mixed or intermediate forms of caries lesion status [2]. This conjoint assessment permits the classification of lesion status according to the most clinical features indicated. Our findings suggest that among non-cavitated caries lesions, the risk of progression is similar if all or most clinical features are positive for active status. This observation corroborates the transitional process of caries arrest.

However, we found some differences in the association of the clinical features of non-cavitated lesions and their progression to cavities. Roughness and loss of luster are biologically related to caries lesion formation/progression because they reflect surface alterations resulting from acid attack and the increase in superficial porosity caused by demineralization. Studies that have demonstrated lustre loss as a classical characteristic associated with active caries have mainly evaluated areas of intense plaque accumulation as areas around orthodontic appliances. In these studies, appliances removal during study permitted the tooth cleaning, and fluoride application was intensified to stimulate quick remineralization of the surface, resulting in a gain in its lustre [4, 33].

Clinically, especially on occlusal surfaces, lesion status's reversion could be slower and less evident than in the conditions mentioned above. In addition, differences in enamel porosity may impede the differentiation of caries lesions and other enamel defects [34]. We should also consider that changes in caries lesions' clinical appearance may have been due to professional cleaning before the examination. However, we believe that this procedure's effect would have been lower because we assessed occlusal surfaces. Accordingly, to predict caries lesion progression, it seems accurate and more straightforward to evaluate only the texture of non-cavitated occlusal caries lesions.

On the other hand, we should consider that texture assessment presents a subjective nature and, in clinical practice, assessing texture may complicate standardization [35]. Alternative models are being proposed for situations involving non-cavitated lesions and may help solve clinicians’ uncertainties, despite requiring the assessment of additional clinical features and more resources. Some important limitations should be raised, especially for the subgroup analysis findings considering the non-cavitated caries lesions. As it was not the study's primary goal, the minimum sample size was not estimated for this purpose. Therefore, we cannot guarantee this analysis may bring adequate statistical power for these analyses. That is why, in some cases, trends were observed in the development cohort and confirmed in the bootstrapped sample. A previous study showed that the time for the non-cavitated lesions in permanent teeth progressing to frank cavitations is longer than for the cavitated ones [36]. We expect the same for primary teeth. Therefore, as we assessed short-term caries progression, these non-cavitated lesions may not have the required time to observe this subsample's events of interest.

As events, we considered lesions that frankly cavitated after 1 year and surfaces that were restored or a tooth that needed to be extracted after this interval. This outcome has been considered a robust outcome to be used in trials [37]. Using it, we expect to identify which situations may lead to real matters to the participants, as needing a dental restoration. As patients were followed up but not treated by the researchers, it is reasonable to consider the restoration as a progression even if another professional had restored the tooth before the established follow-up. Furthermore, we could not eliminate the possibility of interference from individual professionals' choice for operative treatment [38]. A previous study considered this composed outcome, and no major difference in the inferences was observed when restorations were not included in the analysis [8]. Some sound surfaces and initial enamel lesions at baseline were restored during the study, which may not appear as a natural history of caries lesions. However, we observed that a similar proportion of these lesions progressed to advanced lesions (ICDAS scores 5 and 6) after one year, reinforcing the points mentioned above. Therefore, we believe this methodologic option might have slightly impacted the findings presented in this manuscript.

As clinical features tend to reflect caries lesion activity in a fixed time point (the moment of the clinical examination), the importance of conjointly evaluating some features is paramount. However, our findings suggest that some of these parameters could be more helpful in this task than others, simplifying lesion activity assessment and decision-making related to caries lesions. Given the endpoint and the studied predictors, data collection time might not have interfered with the present findings. Changes in young children's caries prevalence has not been observed in the 2000s [39], and relevant modifications would only be expected in a longer time frame.

On the other hand, as we did not validate this prediction model in an independent cohort, further studies should be encouraged in this sense. Such external validation is an essential next step because accurate predictions in our cohort do not necessarily guarantee good accuracy in all patients [40]. Besides, longer-term predictions should also be studied to permit having a more comprehensive view of the decision-making process related to caries lesions in occlusal surfaces of primary teeth and, eventually, determine a rank of prioritization to be applied in clinical practice.

## Conclusions

The presence of cavitations or discontinuities (even with only clinical involvement of the enamel) is a good predictor of short-term caries lesion progression. For non-cavitated lesions, evaluating the enamel roughness is the simplest way to better predict those which will progress. Assessment of these single characteristics may help guide clinicians in clinical decision making. Evaluating other conjoint parameters seems unnecessary for all non-frankly cavitated caries lesions, but in some cases, it may help reduce uncertainty, especially for non-cavitated lesions assessment.

## Supplementary Information

**Additional file 1:** TRIPOD checklist.

**Additional file 2:** Regression equations used for multilevel modelling when testing possible predictors for short-term caries progression (1 year).

**Additional file 3:** Regression coefficients (95% of bias-corrected and accelerated confidence interval) for predictors in univariate models performed with bootstrapped sample.

## Data Availability

Those datasets used and analyzed during the current study, which are not included as Supplementary Material in the published article, are available from the corresponding author on reasonable request.

## Abbreviations

AIC: Akaike information criteria
CI: Confidence interval
dmft: Decayed, filled, missing primary teeth
DMFS: Decayed, filled, missing permanent teeth
ICDAS: International Caries Detection Assessment System
ROC: Receiver operator characteristic
RR: Relative risks
SD: Standard deviation
TRIPOD: Transparent reporting of a prediction model for individual prognosis or diagnosis
