# Supplementary material for: How combining different caries lesions characteristics may be helpful in short-term caries progression prediction: model development on occlusal surfaces of primary teeth
Source: BMC Oral Health. 2021 May 12;21:255. doi: 10.1186/s12903-021-01568-2 (PMC8117278; doi:10.1186/s12903-021-01568-2)
Supplement: Supplementary file 2 — Additional file 2: Regression equations used for multilevel modelling when testing possible predictors for short-term caries progression (1 year). [file 12903_2021_1568_MOESM2_ESM.pdf]

MULTILEVEL POISSON REGRESSION MODELLING

Level 1 Poisson Models (tooth level)

$$\log\mu ij = \beta_{0j} + \beta_{1j}X_{ij} + e_{ij}$$

Level 2 Poisson Models (child level)

$$\beta_{0j} = \gamma_{00} + \gamma_{0i} W_{j} + u_{0j}$$

Poisson Mixed Models:

$$\log\mu ij = (\gamma_{00} + \gamma_{0i} W_{j} + u_{0j}) + \beta_{1j}X_{ij} + e_{ij}$$

i.= tooth 1, 2, …. 733, j= child 1, 2, 3…128

Definitions:

$\log\mu ij$ =dependent variable; β_{0j}: intercept of the dependent variable; β_{1}:slope between the level 1 predictor and the dependent variable; X_{ij}: predictor – level 1; e_{ij}: random errors of prediction for the Level 1 equation, γ_{00}: overall intercept, γ_{0i}:overall regression coefficient, u_{0}: random error component for the deviation of the intercept of a group from the overall intercept, W_{j} :predictor – level 2.

Individual predictors, dummy variables and proposed models.

(considering all predictors are variables in the Level 1 (tooth), we will represent the level 1 regression equation to illustrate modelling)

$$\log\mu ij = \beta_{0j} + \beta_{1j}X_{ij} + e_{ij}$$

Model	X_{ij} – Predictor(s)<br>Lesion characteristic	Type of variable	Categories (n)	Independent Variables (n)	Reference category
#1	Colour (a)	Direct measured variable<br>0: no staining, 1:whitish, 2:yellowish/light brownish, 3:dark brownish/black	4	1	No colour/staining
#2	Presence of lustre (b)	Direct measured variable<br>0: yes, 1: no	2	1	Presence of lustre
#3	Surface integrity (c)	Direct measured variable<br>0: non-cavitated, 1:cavitated	2	1	Non-cavitated surface
#4	Texture (d)	Direct measured variable<br>0: smooth surface, 1: rough surface	2	1	Smooth surface
#5	"Clinical depth" (e)	Direct measured variable<br>0: sound, 1:enamel lesion, 2: dentine lesion	3	1	Sound surface
#6	c0d1[‡]<br>c1d0[‡]<br>c1d1[‡]	Product variable (surface integrity * texture) – [‡]Dummy variables created	-	3	Non-cavitated and smooth surface (c0d0)
#7	c0b1[‡]<br>c1b0[‡]<br>c1b1[‡]	Product variable (surface integrity * lustre) – [‡]Dummy variables created	-	3	Non-cavitated surface with lustre (c0b0)

#8	c0a1[‡]<br>c0a2[‡]<br>c0a3[‡]<br>c1a0[‡]<br>c1a1[‡]<br>c1a2[‡]<br>c1a3[‡]	Product variable (surface integrity * colour) – [‡]Dummy variables created	-	7	Non-cavitated surface with no staining (c0a0)
#9	c0y1[‡]<br>c1y0[‡]<br>c1y1[‡]	Product variable (surface integrity * other signs of activity (y)) – [‡]Dummy variables created	-	3	Non-cavitated surface with no other sign of activity status (c0y0)<br>*y (dummy variable) = have other signs of activity different from surface integrity (y/n)*
#10	d0b1[‡]<br>d1b0[‡]<br>d1b1[‡]	Product variable (texture * presence of lustre) – [‡]Dummy variables created	-	3	Smooth surface with presence of lustre (d0b0)
#11	d0e1[‡]<br>d0e2[‡]<br>d1b0[‡]<br>d1b1[‡]<br>d1e2[‡]	Product variable (texture * “clinical depth”) – [‡]Dummy variables created	-	5	Smooth and sound surface (d0e0)

#12	d0a1‡<br>d0a2‡<br>d0a3‡<br>d1a0‡<br>d1a1‡<br>d1a2‡<br>d1a3		-	7	Smooth surface with no staining (d0a0)
#13	Number of fator combined (f)	Dummy variable created (0: none positive factor, 1: 1 positive factor, 2: at least 2 positive factors)	3	1	None positive factor for activity (f0)
#14	Number of factor combined (g)	Dummy variable created (0: none positive factor, 1, 2: at least 2 positive factors)	2	1	None positive factor for activity (f0)
